# A genetic variant in telomerase reverse transcriptase (*TERT*) modifies cancer risk in Lynch syndrome patients harbouring pathogenic *MSH2* variants

**DOI:** 10.1038/s41598-021-90501-2

**Published:** 2021-05-31

**Authors:** Mariann Unhjem Wiik, Tiffany-Jane Evans, Sami Belhadj, Katherine A. Bolton, Dagmara Dymerska, Shantie Jagmohan-Changur, Gabriel Capellá, Grzegorz Kurzawski, Juul T. Wijnen, Laura Valle, Hans F. A. Vasen, Jan Lubinski, Rodney J. Scott, Bente A. Talseth-Palmer

**Affiliations:** 1 Research Unit, Ålesund Hospital, Møre and Romsdal Hospital Trust, Ålesund, Norway; 2 Hunter Medical Research Institute, New Lambton Heights, Australia; 3 Hereditary Cancer Program, Catalan Institute of Oncology, IDIBELL and CIBERONC, Hospitalet de Llobregat, Barcelona, Spain; 4 School of Biomedical Science and Pharmacy, Faculty of Health and Medicine, University of Newcastle, Newcastle, NSW Australia; 5 Department of Genetics and Pathology, International Hereditary Cancer Center, Pomeranian Medical University, Szczecin, Poland; 6 Department of Human Genetics, Leiden University Medical Center, Leiden, the Netherlands; 7 Department of Gastroenterology and Hepatology, Leiden University Medical Center, Leiden, the Netherlands; 8 Division of Genetics, NSW Health Pathology, John Hunter Hospital, Newcastle, NSW Australia

**Keywords:** Genetics, Cancer genetics

## Abstract

Individuals with Lynch syndrome (LS), have an increased risk of developing cancer. Common genetic variants of *telomerase reverse transcriptase (TERT)* have been associated with a wide range of cancers, including colorectal cancer (CRC) in LS. We combined genotype data from 1881 LS patients, carrying pathogenic variants in *MLH1*, *MSH2* or *MSH6,* for rs2075786 (G>A, intronic variant), 1207 LS patients for rs2736108 (C>T, upstream variant) and 1201 LS patients for rs7705526 (C>A, intronic variant). The risk of cancer was estimated by heterozygous/homozygous odds ratio (OR) with mixed-effects logistic regression to adjust for gene/gender/country of sample origin considering family identity. The AA genotype of SNP rs2075786 is associated with 85% higher odds at developing cancer compared to GG genotype in *MSH2* pathogenic variant carriers (*p* = 0.0160). Kaplan–Meier analysis also shows an association for rs2075786; the AA allele for *MSH2* variant carriers confers risk for earlier diagnosis of LS cancer (log-rank *p* = 0.0011). We report a polymorphism in *TERT* to be a possible modifier of disease risk in *MSH2* pathogenic variant carriers. The rs2075786 SNP in *TERT* is associated with a differential risk of developing cancer for *MSH2* pathogenic variant carriers. Use of this information has the potential to personalise screening protocols for LS patients.

## Introduction

Lynch syndrome (LS) is an autosomal dominantly inherited cancer syndrome that accounts for 2–5% of all colon cancer cases and approximately 2% of all endometrial cancer (EC) cases. LS is characterised by early-onset epithelial cancers in a variety of organs (including but not limited to; CRC, EC, cancer of the kidneys, duodenum, ureter, ovaries, stomach and brain) at a mean age of disease onset that appears to be much lower than that of the general population^1^. LS is a result of pathogenic germline variants in four DNA mismatch repair (MMR) genes (*MLH1*, *MSH2*, *MSH6* and *PMS2*) or by epigenetic inactivation of *MSH2* due to a deletion at the 3’ end of *EPCAM*^2–6^, but an obvious pre-malignant phenotype/genotype correlation is not evident. Genetic variation is the foundation of diversity observed in the human phenotype, and accounts for the large variety of susceptibilities to common diseases^7^. Differences in disease expression are not only observed between patients who harbour causative germline variants in different MMR genes, but also between patients carrying variants in the same gene and even in patients harbouring the same variant^8^, suggesting that other genetic and environmental factors, are likely to be involved in the disease process. The search for genetic variants that have a modifying effect on disease expression has been ongoing but without definitive results^9–12^.

Telomeres are located at the end of chromosomes and have many functions that are critical for genome stability and integrity^13^. Telomere shortening limits the proliferation of normal somatic cells but not cancer cells, which can maintain long telomeres, usually via the enzymatic activity of telomerase^14–16^. Human cancer cells have the capacity of unlimited proliferation potential, associated with the expression of telomerase activity^17^. Telomere shortening has also been shown to result in chromosomal instability^18^. Besides telomere shortening, telomeres may malfunction due to genetic variation in telomere maintenance genes, especially that orchestrated by *telomerase reverse transcriptase (TERT*)^19^. Significant up-regulation of *TERT* is found in Lynch syndrome CRC as well as microsatellite instable sporadic CRC, indicating the influence this gene has on telomere length^20^. The study also shows that mean telomere length systematically shortened in all tumour tissue in LS cancer and sporadic CRC compared to reference systems^20^. Many *TERT* single nucleotide polymorphisms (SNPs) have been associated with a wide range of cancers^21–25^, including CRC^26–28^ and LS cancers^29^. Studies have described polymorphisms that can increase the transcriptional activity of the *TERT* promoter and thus may promote cancer progression^30,31^. Moreover, genome-wide association studies (GWASs) and case–control studies have demonstrated that polymorphisms in *TERT* are associated with various cancers such as skin cancer, CRC and breast cancer^28,32^. While individual *TERT* SNPs have been associated with different types of cancer and telomere length^22,26,33,34^ and a common haplotype with decreased cancer risk substantially reducing *TERT* promoter activity^34^.

*MSH2* deficiency has been shown to lead to accelerated telomere shortening in normal human cells^35^. If a reduction of *MSH2* expression to 50% is sufficient to increase the rate of telomere shortening in normal differentiated tissues in vivo, then there would be an increased risk of early onset of telomere dysfunction leading to cellular senescence in heterozygous carriers that may affect tissue architecture and cancer progression^35^. With telomere shortening being an early event in CRC carcinogenesis, this makes *MSH2* pathogenic variant carriers especially vulnerable.

In this study we have used genotypes of three SNPs in *TERT,* located on chromosome 5p15.33; rs2736108 (upstream variant) and rs7705526 (intronic variant) both of which have been associated with longer telomeres and breast cancer^22^, and rs2075786 (intronic variant) reported to be associated with shorter telomeres and increased cancer risk in LS^29^, using data from four different LS cohorts (two of the cohorts previously described for one of the SNPs in^29^). With additional analysis, we aim to determine if these polymorphisms are associated with the age of cancer onset or cancer risk in this susceptible population. If targeted genetic screening is used to identify patients with further increased risk of developing cancer, more personalized screening strategies may be appropriate to reduce the likelihood of LS patients presenting with cancer.

## Materials and methods

The study complies with the ethical considerations from Hunter New England Research Ethics Committee (Australia), University of Newcastle Human Research Ethics Committee (Australia), the ethics committees of the Pomeranian Academy of Medicine (Poland), ethics committee of Institut d’Investigació Biomèdica de Bellvitge (Spain), Leiden University Medical Centre (the Netherlands) and Regional Committees for Medical and Health Research Ethics (Norway)—all experiments were performed in accordance with institutional guidelines and regulations. Written, informed consent was obtained from all participants. A parent or guardian provided informed consent for participants under the age of 18 years of age.

### Sample cohort

This study consists of 1971 LS patients carrying pathogenic variants (class 4 and 5) in *MLH1*, *MSH2* or *MSH6* (681 Australian, 396 Polish, 240 Spanish and 654 Dutch) from 716 families, representing one of the largest LS cohort published for modifier genes to date. The Spanish and Dutch genotype data for SNP rs2075786 has previously been published^29^, while the two additional SNPs and the Australian and Polish dataset has not. The reason for doing a combined analysis instead of using Australian and Polish samples as a validation cohort is the increased statistical power the larger sample size provides. Note that the statistical power for SNP rs 2075786 is larger than for the two other SNPs due to it being analysed in a larger sample cohort. This study also represents an extension of the previous study with more and deeper analyses. In addition, it has accounted for country in the multi-variable analysis of this study.

### Genotyping

The Australian and Polish LS patient samples were genotyped for SNPs (major > minor allele according to GnomAD) in *TERT*; rs2075786 (G>A), rs2736108 (C>T) and rs7705526 (C>A) using TaqMan SNP assays (Applied Biosystems) for the Australian and Polish sample cohorts. Thermo-cycling was undertaken according to the TaqMan SNP Genotyping Assay Protocol, involving; 10 min at 95 degrees; 40 cycles of 15 s at 95 degrees; and 1 min at 60 degrees. Raw data was generated using the 7500 standard real-time PCR system (Applied Biosystems). Raw data was analysed using TaqMan Genotyper Software (Life Sciences, Foster City, CA).

### Statistical analysis

Statistical analysis was performed using Stata 12.1 (StataCorp LP, TX USA). Pearson’s Chi-square test was used to evaluate deviation from the expected Hardy–Weinberg equilibrium (HWE) and genotype frequency differences between sample cohorts (2 × 3 contingency tables). We applied Bonferroni correction for multiple testing, resulting in a corrected significance threshold of *p* = 0.0167 (0.05 divided by the 3 SNPs tested).

Variation in age of diagnosis between each SNPs genotype was examined using Kaplan–Meier estimator analysis using Wilcoxon’s (Breslow), Log-rank and Tarone-Ware tests to examine homogeneity of the Kaplan–Meier plots. For the Kaplan–Meier analysis, age of diagnosis of LS cancer or CRC is the endpoint for analysis and individuals free from cancer/polyposis were censored at their age at last follow up.

Risk of cancer was estimated for each SNP by genotypic odds ratio (OR) using multilevel mixed-effects logistic regression taking into account family id (because we have both probands and relatives in the cohort), while adjusting for country, gender and gene. Odds ratios, 95% confidence intervals and p-values are presented using forest plots; model coefficients for each model are presented in the supplementary material. Due to significant findings for SNP rs2075786 and to replicate a previous study^29^, additional analysis within *MSH2* carriers was conducted (as described above) but modelling LS cancer diagnosis < 45 years of age, versus those diagnosed after 45 years of age or who were cancer-free with no age restriction.

## Results

There were 1971 samples across four countries (654 samples from the Netherlands and 240 samples from Spain, both previously described^29^, and 681 samples from Australia and 396 samples from Poland) with enough clinical data to warrant inclusion in the current analysis from which 76 samples failed to genotype for all three *TERT* SNPs (sample cohort of 1895).

### Genotyping

Samples from the Netherlands and Spain were originally only genotyped for rs2075786^29^, while the Australian and Polish were genotyped for this SNP for the purpose of the current study. A further 14 samples failed genotyping yielding an analysis cohort of 1881 for this SNP. The Spanish, Australian and Polish samples were genotyped for the two additional SNPs; making 1241 samples available for rs2736108, an additional 34 samples failed genotyping yielding 1207 for analysis, and for rs7705526, 40 samples failed genotyping yielding 1201 samples. Demographic data from combined and individual sample cohorts can be seen in Table 1.

**Table 1:** Demographic data. (A) Displays demographics for the combined LS cohort (rs2075786 and rs2736108 + rs7705526), while (B) Displays demographics for the four countries separately.

A	Samples n	Average age* years	Gender Male (%) / female (%)	Gene *MLH1* (%) / *MSH2* (%) / *MSH6* (%)
**rs2075786 (n = 1895)**				
LS cancer	830	45 (16–81)	351 (42)/479 (58)	363 (44) / 342 (41)/124 (15)*
CRC	632	44 (16–79)	316 (50)/316 (50)	315 (50) / 250 (40)/66 (10)
Cancer-free	1065	42 (11–89)	451 (42)/614 (58)	472 (44) / 363 (34)/230 (22)
**Total**	**1895**			
**rs2736108 and rs7705526 (n = 1241)**				
LS cancer	633	45 (18–84)	266 (42)/367 (58)	292 (46) / 260 (41) / 81 (13)
	503	44 (18–79)	241 (48)/262 (52)	259 (51) / 199 (40) / 45 (9)
Cancer-free	608	40 (12–81)	246 (40)/362 (60)	303 (50) / 205 (34) / 100 (16)
**Total**	**1241**			

B	Sample cohort demographics by country			
	Samples n	Average age* Years (range)	Gender Male (%) / female (%)	Gene *MLH1* (%)/*MSH2* (%)/*MSH6* (%)
LS cancer AU	298	45 (19–81)	173 (58)/125 (42)	106 (36)/136 (46)/56 (18)
LS cancer PL	189	45 (18–71)	121 (64)/68 (36)	101 (54)/74 (39)/14 (7)
LS cancer ES	146	44 (21–79)	73 (50)/72 (50)	85 (58)/50 (34)/11 (8)
LS cancer NL	197	45 (16–78)	112 (57)/85 (43)	71 (36)/82 (42)/43 (22)
**Total**	**803**			
CRC AU	218	44 (19–75)	113 (52)/105 (48)	92 (42)/99 (45)/27 (13)
CRC PL	150	44 (18–69)	86 (58)/64 (42)	85 (57)/55 (37)/10 (6)
CRC ES	135	44 (21–79)	63 (47)/71 (53)	82 (61)/45 (33)/8 (6)
CRC NL	129	44 (16–72)	54 (42)/75 (58)	56 (43)/51 (40)/21 (17)
**Total**	**632**			
Cancer-free AU	321	40 (11–81)	196 (61)/124 (39)	129 (40)/112 (35)/80 (25)
Cancer-free PL	193	39 (13–73)	116 (60)/77 (40)	114 (59)/68 (35)/11 (6)
Cancer-free ES	94	41 (12–82)	50 (53)/44 (47)	60 (64)/25 (26)/9 (10)
Cancer-free NL	457	45 (20–89)	252 (55)/205 (45)	169 (37)/158 (35)/130 (28)
**Total**	**1065**			

* Average age of cancer for cancer patients and average age at last follow up for cancer-free patients.

Table 2 displays genotype frequencies from sample cohorts and as expected there are somewhat significant differences between the cohorts; Australian/Dutch and Polish/Dutch for SNP rs2075786, and Australian/Spanish for SNPs rs2736108 and rs7705526 (all *p* > 0.03). For SNP rs2736108 the Australian genotype frequency is significantly different from the Polish (*p* = 0.001).

**Table 2:** Genotype frequencies in the combined sample cohort and for each of the four countries included.

TERT SNP	Combined sample cohort Total n (%)	Australian sample cohort Total n (%)	Polish sample cohort Total n (%)	Spanish sample cohort Total n (%)	Netherland sample cohort Total n (%)
**rs2075786**	1881	608	380	239	654
GG	782 (41.6)	228 (37.5)	155 (40.8)	109 (45.6)	290 (44.3)
GA	852 (45.3)	296 (48.6)	162 (42.6)	102 (42.7)	292 (44.7)
AA	247 (13.1)	84 (13.9)	63 (16.6)	28 (11.7)	72 (11.0)
**rs2736108**	1207	601	381	225	
CC	629 (52.1)	279 (46.4)	222 (58.3)	128 (56.9)	
CT	475 (39.4)	261 (43.4)	133 (34.9)	81 (36.0)	
TT	103 (8.5)	61 (10.2)	26 (6.8)	16 (7.1)	
**rs7705526**	1201	601	375	225	
CC	513 (42.7)	280 (46.5)	146 (38.9)	87 (38.7)	
CA	549 (45.7)	263 (43.8)	182 (48.6)	104 (46.2)	
AA	139 (11.6)	58 (9.7)	47 (12.5)	34 (15.1)	

### Statistical analysis—risk of LS cancer

Both simple logistic regression and Kaplan–Meier analysis show that the patient cohort is consistent with published literature; carriers of *MSH6* pathogenic variants have a decreased risk of LS cancer compared to *MLH1* pathogenic variant carriers (OR = 0.68, CI = 0.50–0.92, p = 0.014), and later age of onset compared *MLH1* and *MSH2* variants carriers, see supplementary Fig. S1. None of the three *TERT* SNPs demonstrated independent associations with risk of LS cancer or age of diagnosis (i.e. when not accounting for LS mutant gene); observed genotype frequencies, crude genotypic odds ratios and odds ratios are presented in supplementary table 1. There were no deviations from HWE (rs2075786 *p* = 0.53, rs2736108 *p* = 0.32, rs7705526 *p* = 0.67).

**Figure 1:**
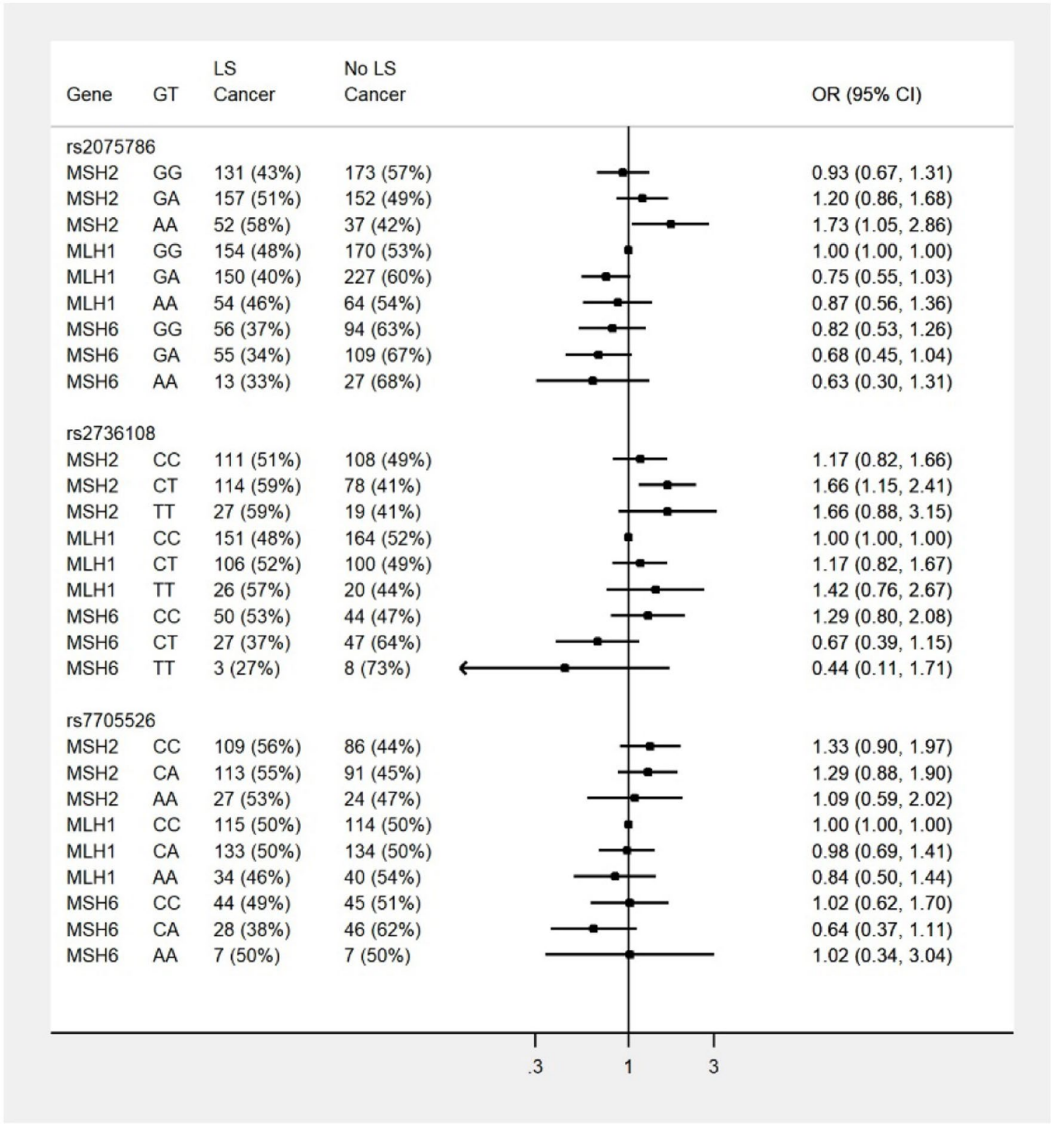
This forest plot displays across cohort, odds ratios for risk of LS cancer by gene and genotype for the three *TERT* SNPs in the current study, the reference group is *MLH1* major genotype. The reference group for each model, is the gene/genotype group set to unity. All other ORs are relative to this reference group.

Results for the mixed-effects logistic regression investigating the interactions of each SNP with the respective gene, adjusting for confounders, are presented in Fig. 1; depicted are the corresponding odds ratios by each level of gene and genotype with the reference group (*MLH1* homozygous major allele) set at unity. There was weak evidence that effects for rs2075786 and rs2736108 genotypes were different by gene (p for interaction = 0.07 and 0.05, respectively). Model coefficients, confidence intervals and p-values are presented in supplementary tables S2-S4.

For SNP rs2075786 in Fig. 1, it was apparent that within *MLH1* and *MSH6* variant carriers, the genotype risk patterns were similar; the GG genotype confers the greatest risk but it was not statistically significantly different from the other genotypes (95% confidence intervals overlap substantially). Whereas for *MSH2* variant carriers, harbouring the heterozygous genotype was associated with greater risk, and those homozygous for A had the greatest risk of cancer across the cohort.

**Figure 2:**
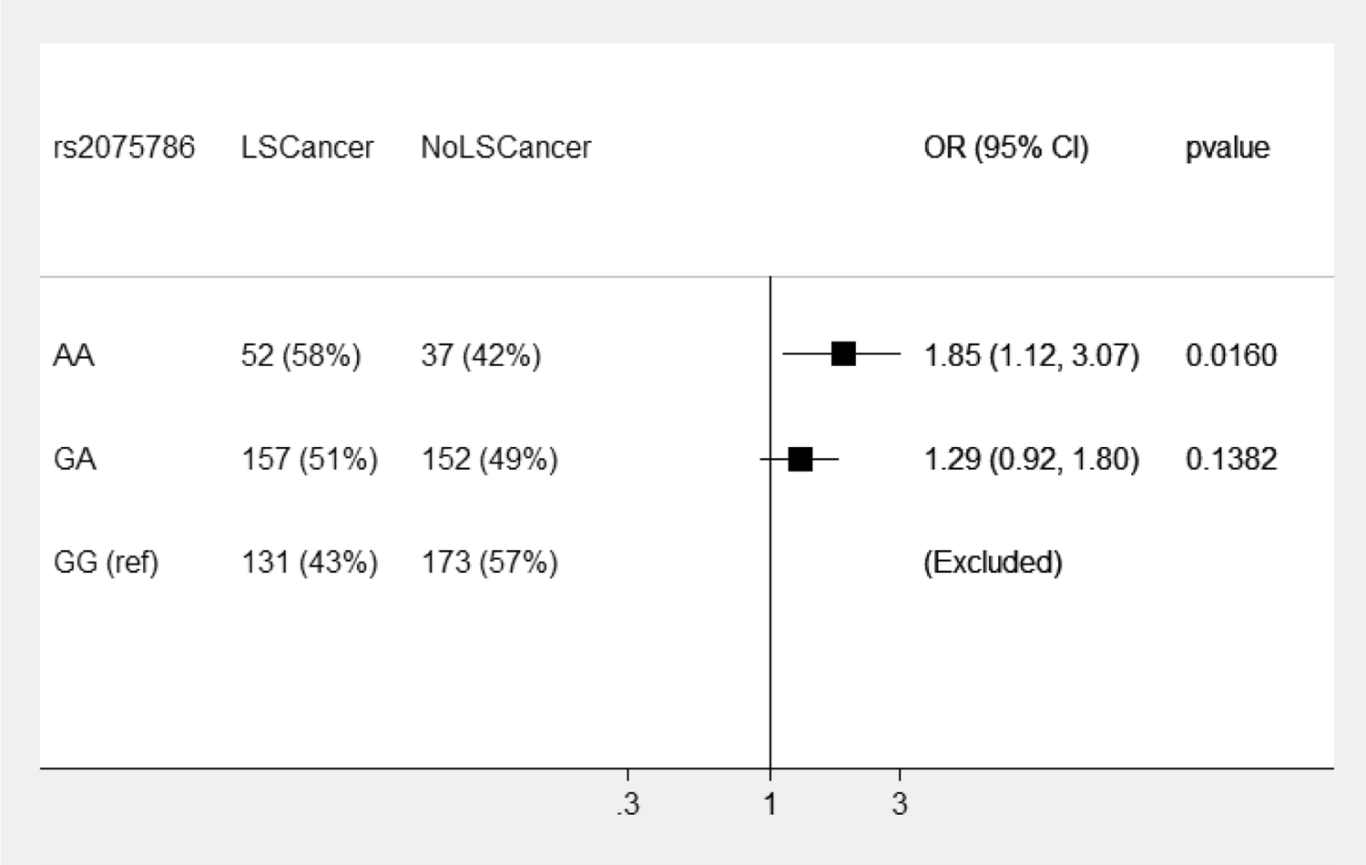
Displays rs2075786 odds ratios for risk of LS cancer in *MSH2* pathogenic variant carriers. The reference is genotype GG (wildtype).

Within *MSH2* variant carriers, the AA genotype of rs2075786 is associated with 85% higher odds of developing cancer compared to *MSH2* carriers with the GG genotype (Fig. 2, ORs within *MSH2* pathogenic variant carriers only, estimated from the same model as Fig. 1).

**Figure 3:**
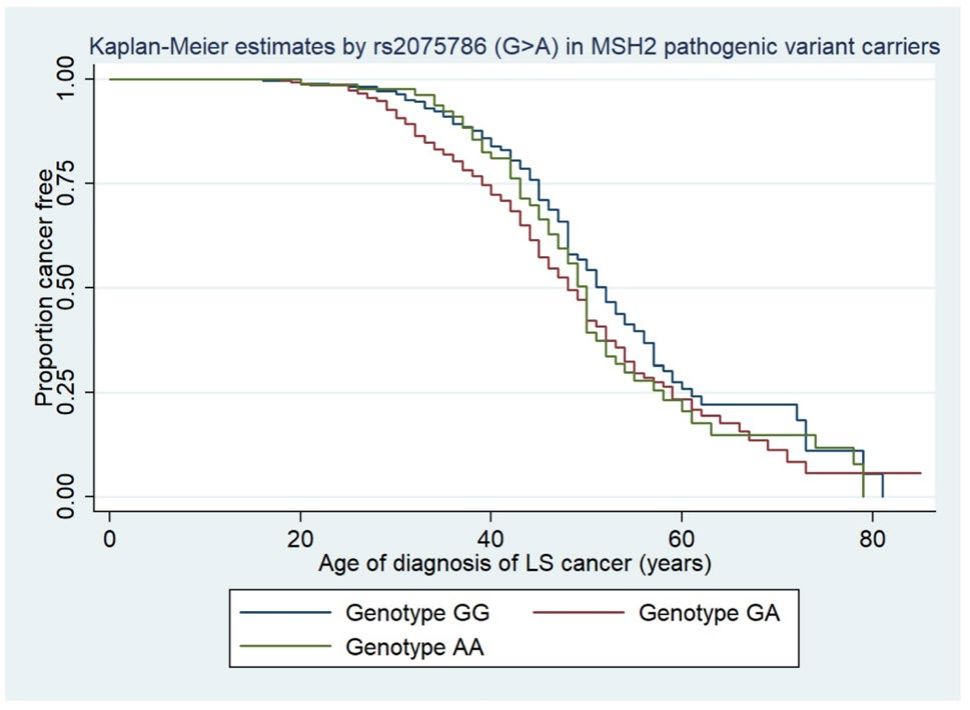
Kaplan–Meier estimated by rs2075786 genotypes in *MSH2* pathogenic variant carriers. The graph shows the effect the genotypes has on age of diagnosis of Lynch Syndrome (LS) cancer in LS patients. A significant difference in the age of diagnosis of LS cancer can be seen between the three genotypes (Log-rank p = 0.0011, Wilcoxon p = 0.0006 and Tarone-Ware p = 0.0007). LS patients over the age of 50 years and carriers of the A allele (GA and AA genotype) will develop LS cancer earlier than LS patients carrying the GG genotype will.

A previous study of LS patients with *MSH2* variants, linked SNP rs2075786 to an increased risk of cancer diagnosis younger than 45 years age using logistic regression^34^. When we analysed this outcome we also observed an association (see supplementary Fig. S2); the heterozygous genotype (GA) is linked to the greatest risk of cancer (OR 1.79, 95% CI 1.2 to 2.7, p = 0.005; Model coefficients, confidence intervals and p- values are presented in supplementary table S5). Our Kaplan–Meier analysis also demonstrated that *MSH2* variant carriers who also carry the rs2075786 A allele have earlier onset of cancer (Kaplan–Meier analysis log-rank p = 0.0011, Wilcoxon p = 0.0006 and Tarone-Ware p = 0.0007 see Fig. 3), which was not observed for *MLH1* and *MSH6* variant carriers (log-rank p = 0.3524 and p = 0.3763, respectively).

**Figure 4:**
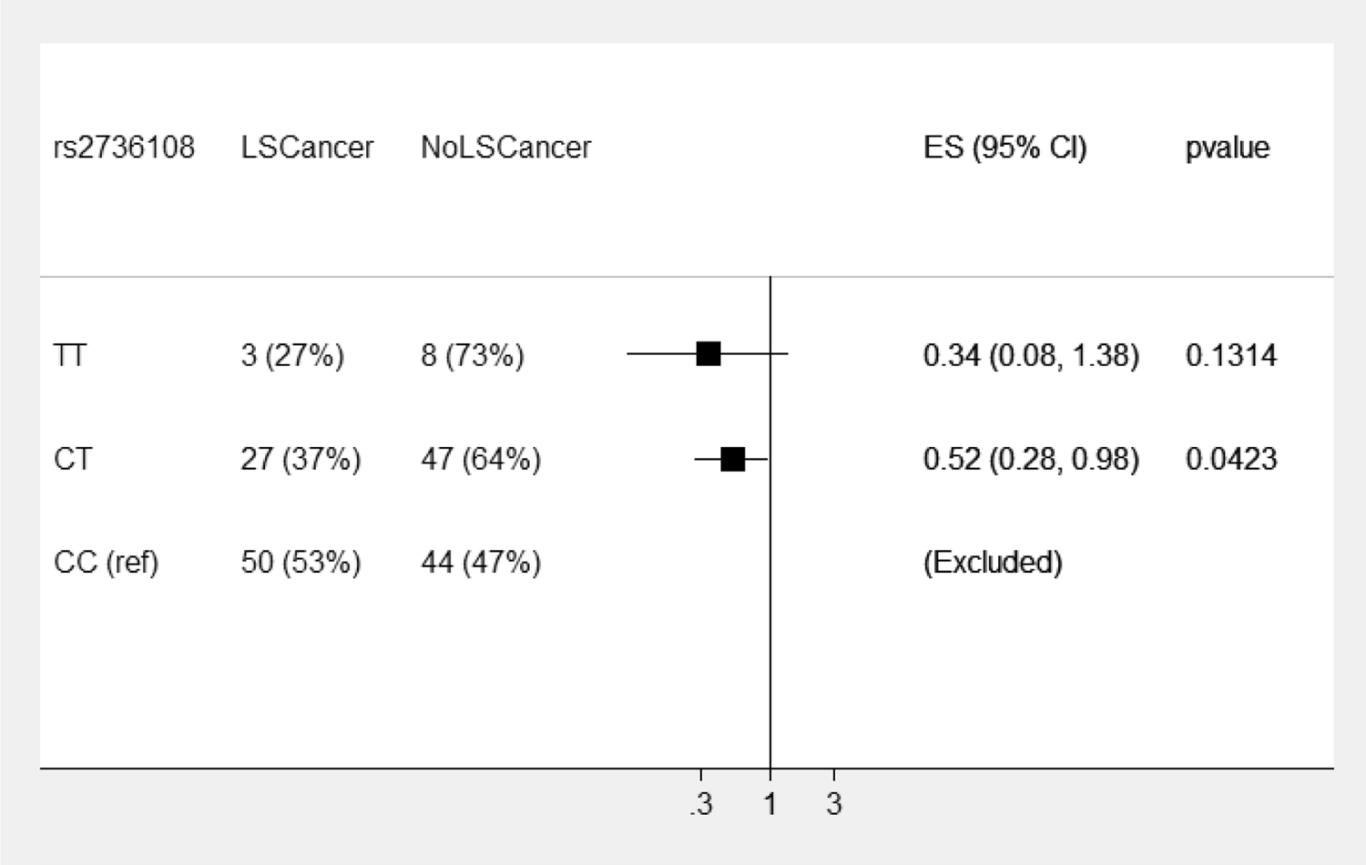
Displays rs2736108 odds ratios for risk of LS cancer in *MSH6* pathogenic variant carriers. The reference is genotype CC.

For rs2736108, the risk patterns observed for *MLH1* variant carriers was similar to *MSH2* variant carriers whereas, the pattern for *MSH6* variant carriers was different (see Fig. 1). Within *MSH6* variant carriers, the C allele confers greater risk than the T allele (Fig. 4 presents the odds ratios for the genotypes). The odds of LS cancer were 48% lower for the CT genotype vs the CC genotype (OR 0.52 95%CI 0.29 to 098) and the odds for the TT genotype were lower again however the confidence interval overlapped one due to the small number of observations with this covariate pattern.

There was a lack of evidence that the risk pattern for genotypes of rs7705526 differed by gene (p for interaction = 0.73).

## Discussion

Here we present results from a large sample cohort of 1881 LS patients that statistically show that a polymorphism in *TERT* influences disease risk in LS patients. SNP rs2075786 in *TERT* modifies cancer risk in LS patients with mutations in *MSH2*, the variant AA genotype is associated with 85% higher odds of cancer compared to the wildtype GG genotype.

The current study was undertaken as cancer-affected LS patients with the AA genotype of SNP rs2075786 had been shown to have shorter telomeres than those with GG genotype and the A allele was marginally associated with LS cancer in patients < 45 years of age^29^. In silico analysis predicted the A allele of SNP rs2075786 eliminates a retinoid binding site, causing natural retinoids not to efficiently limit *TERT* expression, culminating in accelerated tumour growth^29^. This finding is consistent with another study that revealed leukocyte telomeres of patients with LS cancer were shorter than those of controls and unaffected LS patients^36^, suggesting that shortened telomeres are a result of the disease or an additional risk factor for LS patients. A second LS study reported no evidence of association between *TERT* SNPs and risk of CRC, overall or when stratified by gender and MMR gene after adjustment for multiple testing and censored by age 45 years^37^, but differently to the current study they only considered CRC risk not including all LS associated cancers. Another advantage of the current study is the large sample size and the ability to detect smaller effect sizes.

It has been shown that cell lines with variants in MMR genes show telomere instability, with highest mutation frequency in *MSH2* deficient cells^35^. Reduction in *MSH2* expression leads to accelerated telomere shortening in normal cells^35^ and *MSH2* deficient cells have been shown to have minor telomere capping effects^38^. *MSH2* is associated with the *TERT* promoter and regulates promoter activity, i.e. knockdown of *MSH2* results in a significant reduction of telomerase activity in human oral squamous cell carcinoma cells^39^. *MSH2* variants lead to accelerated telomere shortening in normal cells (an early event in CRC carcinogenesis) and the A allele of SNP rs2075786 is predicted to cause early telomerase activation (carriers of the AA genotype have shorter telomeres^29^). Individually they might just have subtle inhibitory effect on *TERT* but together they may increase LS patients’ risk of cancer development. This can explain why we observe the significantly increased risk of cancer in LS patients compared to LS cancer-free patients and makes SNP rs2075786 a plausible modifier for disease risk in *MSH2* pathogenic variant carriers.

Possible biases in the current study include confounding factors such as lifestyle, smoking and other environmental factors influencing the reported results, however since there was no specific selection for patients these variables are likely to be equally distributed across the patient cohort. Studies on modifier genes in LS are difficult due to all the variables affecting cancer risk, and controversial results have rather been the rule than the exception, but with increased sample sizes we are now hoping to avoid this. Ascertainment bias related to sampling and selection bias (where some members are less likely to be included than others) should not be a problem since there is good representation of both cancer affected and unaffected MMR variant carriers. Our results could aid in explaining the controversial evidence for anticipation in LS^40,41^, even though we have not looked into this in the current study, as increased rate of telomere shortening in *MSH2* deficient cells provides a mechanism that may contribute to genetic anticipation in some LS families^35^. We were unable to control for differences in individual causal germline variants (i.e. frameshift, splice site, etc.) in individual genes as this information was not available for all patients.

In conclusion, we present a polymorphism in *TERT* to be a possible modifier of disease risk in *MSH2* pathogenic variant carriers. The rs2075786 SNP in *TERT* is associated with a differential risk of developing cancer for *MSH2* pathogenic variant carriers. By including this SNP in future risk algorithms, it should be possible to tailor surveillance options for individual patients. Use of this information has the potential to personalize screening protocols for LS patients.

## Supplementary Information

Supplementary Information.
