# Supplementary material for: A genetic variant in telomerase reverse transcriptase (TERT) modifies cancer risk in Lynch syndrome patients harbouring pathogenic MSH2 variants
Source: Sci Rep. 2021 May 31;11:11401. doi: 10.1038/s41598-021-90501-2 (PMC8166931; doi:10.1038/s41598-021-90501-2)
Supplement: Supplementary file 1 — Supplementary Information. [file 41598_2021_90501_MOESM1_ESM.docx]

**A genetic variant in telomerase reverse transcriptase (TERT) modifies cancer risk in Lynch syndrome patients harbouring pathogenic MSH2 variants**

Mariann Unhjem Wiik^1^, Tiffany-Jane Evans^2^, Sami Belhadj^3^, Katherine A. Bolton^4^, Dagmara Dymerska^5^, Shantie Jagmohan-Changur^6^, Gabriel Capellá^3^, Grzegorz Kurzawski^5^, Juul T. Wijnen^6^, Laura Valle^3^, Hans F.A Vasen^7^, Jan Lubinski^5^, Rodney J. Scott^2,4,8^ and Bente A. Talseth-Palmer^1,2,4^

^1^ Research Unit, Ålesund Hospital, Møre and Romsdal Hospital Trust, Norway

^2^ Hunter Medical Research Institute, New Lambton Heights, Australia

^3^ Hereditary Cancer Program, Catalan Institute of Oncology, IDIBELL and CIBERONC, Hospitalet de Llobregat, Barcelona, Spain

^4^ School of Biomedical Science and Pharmacy, Faculty of Health and Medicine, University of Newcastle, Newcastle, Australia

^5^ International Hereditary Cancer Center, Department of Genetics and Pathology, Pomeranian Medical University, Szczecin, Poland

^6^ Department of Human Genetics, Leiden University Medical Center, Leiden, the Netherlands

^7^ Department of Gastroenterology and Hepatology, Leiden University Medical Center, Leiden, the Netherlands

^8^ Division of Genetics, NSW Health Pathology, John Hunter Hospital, Newcastle, NSW, Australia

**Corresponding author**

Dr Bente Talseth-Palmer
University of Newcastle
Newcastle, NSW 2308
AUSTRALIA
Email: bente.talseth-palmer@newcastle.edu.au

**Figure S1** Kaplan–Meier estimated by mutated gene in the total sample cohort. The graph shows the effect the mutated gene has on age of diagnosis of Lynch Syndrome (LS) cancer in LS patients. A significant difference (Log‐rank/Wilcoxon/Tarone-Ware *p* ≤ 0.0001) in the age of diagnosis of LS cancer can be seen between individuals with *MLH1* pathogenic variants (52 years), *MSH2* pathogenic variants (50 years) and *MSH6* pathogenic variants (61 years). The number of years are extracted where 50% of the population is cancer free.

**
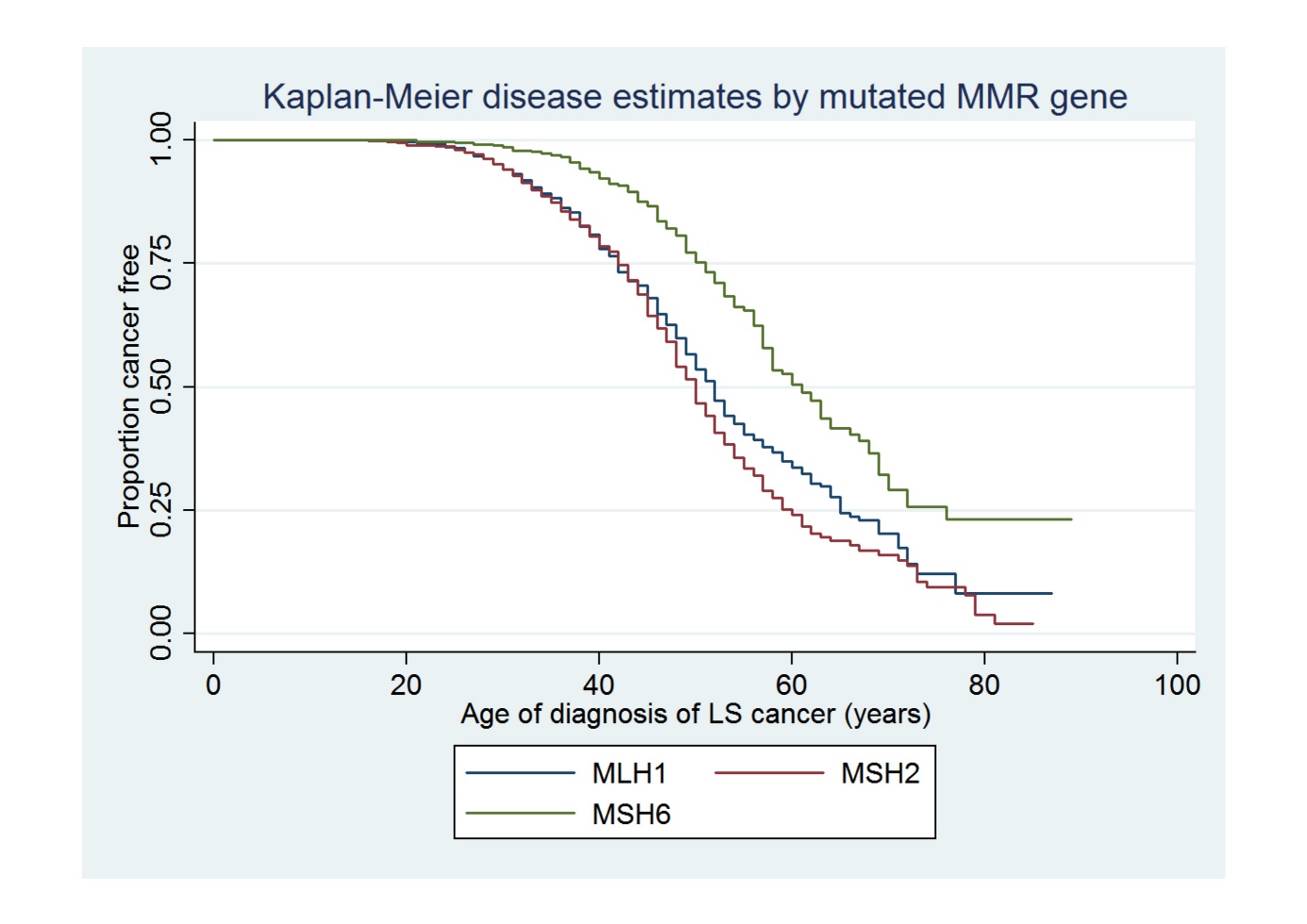
**

**Figure S2** Displays rs2075786 odds ratios for risk of LS cancer <45 years of age in *MSH2* pathogenic variant carriers.

**
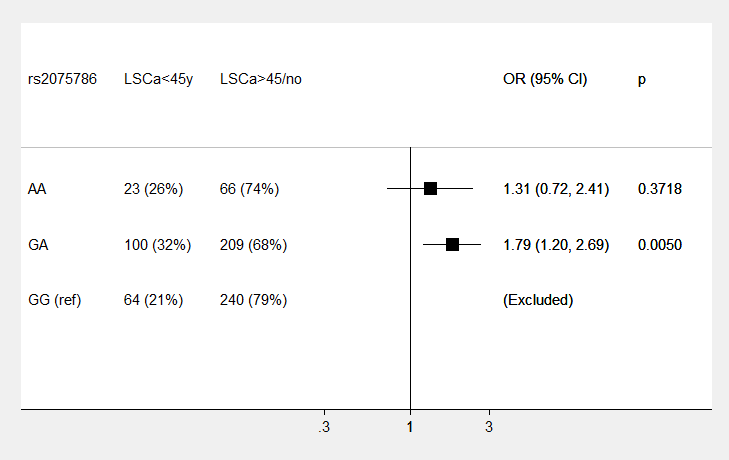
**

**Table S1** Crude and adjusted genotype odds ratios for Lynch syndrome (LS) cancer within the sample cohort across all genes.

| TERT SNP | Total  n (%) | LS cancer free  n (%) | LS cancer  n (%) | Crude mixed-effects logistic regression | | Adjusted mixed-effects logistic regression* | | Test equality of survivor functions  Log-rank test *p*-value |
| --- | --- | --- | --- | --- | --- | --- | --- | --- |
| rs2075786 | 1881 | 1057 | 824 | OR (CI) | p |  | p | 0.9456 |
| GG | 782 (41.6) | 439 (42.5) | 343 (41.6) | 1.0 | 0.440 | 1.0 | 0.465 |  |
| GA | 852 (45.3) | 490 (46.4) | 362 (43.9) | 0.95 (0.77-1.18) |  | 0.94 (0.76-1.16) |  |  |
| AA | 247 (13.1) | 128 (12.1) | 119 (14.4) | 1.16 (0.85-1.60) |  | 1.13 (0.84-1.54) |  |  |
|  |  |  |  |  |  |  |  |  |
| rs2075786 <45 years^*^ | 1881 | 1452 | 429 | OR (CI) | p |  | p | 0.660 |
| GG | 782 (42.6) | 610 (42.0) | 172 (40.1) |  | 0.8131 |  | 0.8023 |  |
| GA | 852 (45.3) | 654 (45.0) | 198 (46.2) | 1.09 (0.84-1.41) |  | 1.09 (0.85-1.40) |  |  |
| AA | 247 (13.1) | 188 (13.0) | 59 (13.8) | 1.03 (0.71-1.51) |  | 1.05 (0.73-1.52) |  |  |
|  |  |  |  |  |  |  |  |  |
| rs2736108 | 1207 | 592 | 615 | OR (CI) | p |  | p | 0.6466 |
| CC | 629 (52.1) | 317 (53.5) | 312 (50.7) |  | 0.575 | 1.0 | 0.482 |  |
| CT | 475 (39.4) | 228 (38.5) | 247 (40.2) | 1.10 (0.86-1.40) |  | 1.13 (0.88-1.44) |  |  |
| TT | 103 (8.5) | 47 (8.0) | 56 (9.1) | 1.21 (0.80-1.84) |  | 1.23 (0.8-1.89) |  |  |
|  |  |  |  |  |  |  |  |  |
| rs7705526 | 1201 | 591 | 610 | OR (CI) | p |  | p | 0.0572 |
| CC | 513 (42.7) | 245 (41.5) | 268 (43.9) | 1.0 | 0.677 | 1.0 | 0.630 |  |
| CA | 549 (45.7) | 275 (46.5) | 274 (44.9) | 0.91 (0.72-1.17) |  | 0.92 (0.72-1.18) |  |  |
| AA | 139 (11.6) | 71 (12.0) | 68 (11.1) | 0.87 (0.60-1.27) |  | 0.84 (0.57-1.24) |  |  |

*adjusting for country and gender

**Table S2** Mixed-effects logistic regression results for the interaction of SNP rs2075786 with gene mutation. Results adjust for confounding by gender and country.

| Variable | Coefficient (95%CI) | Type1 p | Type 3 p |
| --- | --- | --- | --- |
| rs2075786(ref=GG) |  |  |  |
| GA | -0.28 (-0.60,0.03) | 0.078 | 0.658 |
| AA | -0.14 (-0.58,0.31) | 0.551 |  |
| Gene (ref= MLH1) |  |  |  |
| MSH2 | -0.07 (-0.41,0.27) | 0.692 | 0.001 |
| MSH6 | -0.20 (-0.63,0.23) | 0.357 |  |
| rs2075786#Gene |  |  |  |
| GA#MSH2 | 0.54 (0.08,1.00) | 0.022 | 0.073 |
| GA#MSH6 | 0.10 (-0.48,0.69) | 0.725 |  |
| AA#MSH2 | 0.75 (0.08,1.43) | 0.028 |  |
| AA#MSH6 | -0.12 (-1.01,0.77) | 0.792 |  |
| Gender (ref=female) |  |  |  |
| Male | 0.00 (-0.19,0.20) | 0.983 | 0.983 |
| Country (ref=Australia) |  |  |  |
| Poland | 0.04 (-0.24,0.31) | 0.802 | 0.000 |
| Spain | 0.58 (0.25,0.92) | 0.001 |  |
| Netherlands | -0.77 (-1.03,-0.52) | 0.000 |  |

**Table S3** Mixed-effects logistic regression results for the interaction of SNP rs2736108 with gene mutation. Results adjust for confounding by gender and country.

| Variable | Coefficient (95%CI) | Type1 p | Type 3 p |
| --- | --- | --- | --- |
| rs2736108 (ref=CC) |  |  |  |
| CT | 0.15 (-0.20,0.51) | 0.4003 | 0.8814 |
| TT | 0.35 (-0.28,0.98) | 0.2753 |  |
| Gene (ref=MLH1) |  |  |  |
| MSH2 | 0.15 (-0.20,0.51) | 0.3913 | 0.0342 |
| MSH6 | 0.26 (-0.22,0.73) | 0.2919 |  |
| rs2736108#Gene |  |  |  |
| CT#MSH2 | 0.20 (-0.33,0.74) | 0.4627 | 0.0574 |
| CT#MSH6 | -0.80 (-1.52,-0.08) | 0.0288 |  |
| TT#MSH2 | 0.00 (-0.90,0.91) | 0.9926 |  |
| TT#MSH6 | -1.43 (-2.96,0.11) | 0.0682 |  |
| Gender (ref=female) |  |  |  |
| Male | 0.08 (-0.15,0.32) | 0.4917 | 0.4917 |
| Country (ref=Australia) |  |  |  |
| Poland | 0.02 (-0.25,0.29) | 0.8691 | 0.048 |
| Spain | 0.40 (0.07,0.73) | 0.0182 |  |

**Table S4** Mixed-effects logistic regression results for the interaction of SNP rs7705526 with gene mutation. Results adjust for confounding by gender and country.

| Variable | Coefficient (95%CI) | Type1 p | Type 3 p |
| --- | --- | --- | --- |
| rs7705526 (ref=CC) |  |  |  |
| CA | -0.02 (-0.38,0.34) | 0.9267 | 0.4782 |
| AA | -0.17 (-0.70,0.36) | 0.5313 |  |
| Gene (ref=MLH1) |  |  |  |
| MSH2 | 0.28 (-0.11,0.68) | 0.1591 | 0.1523 |
| MSH6 | 0.02 (-0.48,0.53) | 0.9313 |  |
| rs7705526#Gene |  |  |  |
| CA#MSH2 | -0.01 (-0.55,0.53) | 0.9756 | 0.7325 |
| CA#MSH6 | -0.45 (-1.18,0.28) | 0.228 |  |
| AA#MSH3 | -0.03 (-0.85,0.80) | 0.948 |  |
| AA#MSH6 | 0.17 (-1.10,1.43) | 0.7962 |  |
| Gender (ref=female) |  |  |  |
| Male | 0.08 (-0.16,0.31) | 0.5133 | 0.5133 |
| Country (ref=Australia) |  |  |  |
| Poland | 0.01 (-0.27,0.28) | 0.9673 | 0.0303 |
| Spain | 0.43 (0.09,0.76) | 0.0127 |  |

**Table S5** Outcome = LS cancer dx <=45 years of age. Mixed-effects logistic regression results for the interaction of SNP rs2075786 with gene mutation. Results adjust for confounding by gender and country.

| Variable | Coefficient (95%CI) | Type1 p | Type 3 p |
| --- | --- | --- | --- |
| rs2075786(ref=GG) |  |  |  |
| GA | -0.25 (-0.61,0.11) | 0.1753 | 0.9415 |
| AA | -0.12 (-0.63,0.38) | 0.6336 |  |
| Gene (ref= MLH1) |  |  |  |
| MSH2 | -0.27 (-0.66,0.13) | 0.1849 | 0.0001 |
| MSH6 | -0.89 (-1.48,-0.31) | 0.0029 |  |
| rs2075786#Gene |  |  |  |
| GA#MSH2 | 0.81 (0.28,1.33) | 0.0028 | 0.0309 |
| GA#MSH6 | -0.12 (-0.97,0.72) | 0.7723 |  |
| AA#MSH2 | 0.39 (-0.38,1.15) | 0.322 |  |
| AA#MSH6 | 0.15 (-1.05,1.36) | 0.8035 |  |
| Gender (ref=female) |  |  |  |
| Male | 0.09 (-0.15,0.32) | 0.4657 | 0.4657 |
| Country (ref=Australia) |  |  |  |
| Poland | -0.12 (-0.44,0.21) | 0.4857 | 0.0000 |
| Spain | 0.61 (0.24,0.98) | 0.0014 |  |
| Netherlands | -0.64 (-0.96,-0.32) | 0.0001 |  |
